# Adipogenic and energy metabolism gene networks in *longissimus lumborum *during rapid post-weaning growth in Angus and Angus × Simmental cattle fed high-starch or low-starch diets

**DOI:** 10.1186/1471-2164-10-142

**Published:** 2009-03-31

**Authors:** Daniel E Graugnard, Paola Piantoni, Massimo Bionaz, Larry L Berger, Dan B Faulkner, Juan J Loor

**Affiliations:** 1 Mammalian NutriPhysioGenomics, Department of Animal Sciences, University of Illinois, Urbana, Illinois, 61801, USA; 2 Division of Nutritional Sciences, University of Illinois, Urbana, Illinois, 61801, USA; 3 Department of Animal Sciences, University of Illinois, Urbana, Illinois, 61801, USA

## Abstract

**Background:**

Transcriptional networks coordinate adipocyte differentiation and energy metabolism in rodents. The level of fiber and starch in diets with adequate energy content fed to young cattle has the potential to alter intramuscular adipose tissue development in skeletal muscle. Post-weaning alterations in gene expression networks driving adipogenesis, lipid filling, and intracellular energy metabolism provide a means to evaluate long-term effects of nutrition on longissimus muscle development across cattle types.

**Results:**

*Longissimus lumborum *(LL) from Angus (n = 6) and Angus × Simmental (A × S; n = 6) steer calves (155 ± 10 days age) fed isonitrogenous high-starch (HiS; 1.43 Mcal/kg diet dry matter; n = 6) or low-starch (LoS; 1.19 Mcal/kg diet dry matter; n = 6) diets was biopsied at 0, 56, and 112 days of feeding for transcript profiling of 31 genes associated with aspects of adipogenesis and energy metabolism. Intake of dietary energy (9.44 ± 0.57 Mcal/d) across groups during the study did not differ but feed efficiency (weight gain/feed intake) during the first 56 days was greater for steers fed HiS. Expression of *PPARG *increased ca. 2-fold by day 56 primarily due to HiS in A × S steers. Several potential *PPARG*-target genes (e.g., *ACACA*, *FASN*, *FABP4*, *SCD*) increased 2.5-to-25-fold by day 56 across all groups, with responses (e.g., *FASN*, *FABP4*) being less pronounced in A × S steers fed LoS. This latter group of steers had markedly greater blood plasma glucose (0.99 vs. 0.79 g/L) and insulin (2.95 vs. 1.17 μg/L) by day 112, all of which were suggestive of insulin resistance. Interactions were observed for *FABP4*, *FASN*, *GPAM*, *SCD*, and *DGAT2*, such that feeding A × S steers high-starch and Angus steers low-starch resulted in greater fold-changes by day 56 or 112 (*GPAM*). Marked up-regulation of *INSIG1 *(4-to-8-fold) occurred throughout the study across all groups. *SREBF1 *expression, however, was only greater on day 112 namely due to LoS in A × S steers. The lipogenic transcription factor *THRSP *was 6-to-60-fold greater by day 56 primarily due to HiS in A × S steers, constituting the greatest response among all genes.

**Conclusion:**

Results involving gene markers of mature adipocytes (e.g., *PPARG*, *THRSP*, *SCD*) provided evidence of intramuscular adipose tissue differentiation during the early portion of the growing phase. The resulting gene networks underscored a central role for *PPARG *in controlling transcription of genes which are known to co-ordinately regulate adipocyte differentiation and lipid filling in non-ruminants. Unlike rodents, *INSIG1 *appears to play an important role in cattle muscle adipogenesis. We propose that a network of transcription regulators and nuclear receptors including *PPARG*-target genes,* INSIG1*, and *THRSP*, coordinate activation of adipocyte differentiation and lipid filling at an early age.

## Background

Most biological traits are complex, i.e. they are under the control of an interacting network of genes, each with a small effect, and of environmental factors such as nutrition [1]. Metabolic regulation in complex organisms relies partly on transcriptional control as a long-term mechanism affecting the level of expression of key enzymes [2]. In rodents, there is high correlation between mRNA expression of target genes and recruitment of lipogenic transcription factors or nuclear receptors and their co-regulatory proteins to promoter regions, suggesting that gene expression analysis is useful for inferring transcriptional activity [3]. Transcriptional regulation of hepatic lipogenic gene expression, adipogenesis, and skeletal muscle fatty acid oxidation in rodents is under control of sterol regulatory element binding factor 1 (*SREBF1*) [4] and the ligand-activated nuclear receptors PPARγ (*PPARG*) and PPARδ (*PPARD*) [5,6]. Less is known regarding the molecular events during skeletal muscle growth in livestock species such as cattle and pigs [7]. However, recent work has begun to explore large-scale transcriptomic adaptations in skeletal muscle of cattle in response to plane of nutrition (e.g., normal vs. underfeeding) or age [8-11]. Although mRNA expression is one of multiple factors to be considered when studying the complex molecular networks working simultaneously in tissues of varying cell types (e.g., myocytes, non-differentiated stem cells, pre-adipocytes, adipocytes [7]) like skeletal muscle, it provides valid information to aid in designing more detailed functional studies.

Young steer calves are relatively more efficient at converting nutrients to muscle gain [12,13]. Weaning calves at an earlier age than the conventional 205 days is a management practice that has shown to enhance growth rate, modify carcass composition, and modify meat quality [14-16]. Exposing them to high-starch diets at an early vs. conventional age could increase the likelihood that they reach their genetic potential to marble (i.e., deposit intramuscular fat). To our knowledge, no studies have been conducted to examine the influence of dietary starch level at an early age on gene networks regulating pivotal pathways for desired phenotypes of economically-important traits. High-starch/low-fiber diets, through shifting the pattern of end-products of ruminal fermentation towards greater propionate, provide readily-available sources of energy (i.e., glucose) for growing muscle in early-weaning management systems [12,13]. A previous study with early-weaned Angus × Simmental steers reported greater intramuscular fat and backfat thickness at the end of the growing phase in animals fed a high-starch vs. high-fiber diet [17]. Energy available for gain was ca. 35% greater in steers fed the high-starch diet, which undoubtedly contributed to enhanced fat deposition.

The central hypotheses of the present study were that adipogenic and energy metabolism gene networks in *longissimus lumborum *(**LL**) muscle tissue during rapid post-weaning growth would be altered to different extents by genotype as well as feeding diets that varied in level of starch but provided similar amounts of energy for gain. Thus, specific objectives were to study mRNA expression of selected genes associated with insulin signaling and glucose transport, fatty acid uptake and activation, intracellular fatty acid transport, de novo fatty acid synthesis, esterification, desaturation, transcriptional regulation of adipogenesis and differentiation, and energy metabolism (Table 1). These genes potentially compose a large interactive network [6,7] controlling metabolism in cells of cattle LL tissue.

**Table 1:** Description of genes analyzed in skeletal muscle tissue.

Gene symbol^1^	Cellular localization	Biological process
*ACACA*	Cytoplasm	Fatty acid biosynthesis
*ACLY*	Cytosol	Citrate metabolic process
*ACSL1*	Plasma and ER membranes	Fatty acid metabolism
*AGPAT1*	ER membrane	Phosphatidic acid biosynthesis
*CD36*	Plasma membrane	Fatty acid metabolism
*DGAT1*	ER membrane	Triacylglycerol metabolism
*DGAT2*	ER membrane	Triacylglycerol metabolism
*FABP4*	Cytosol	Fatty acid binding, transport
*FADS2*	ER membrane	Unsaturated fatty acid synthesis
*FASN*	Cytosol	Fatty acid biosynthesis
*G6PD*	Cytosol	Pentose-phosphate shunt
*GPAM*	Cytosol, mitochondria	Triacylglycerol biosynthesis
*INSIG1*	ER membrane	Lipid metabolism, cell proliferation
*INSR*	Plasma membrane	Insulin receptor signaling
*IRS1*	Cytosol	Insulin receptor signaling
*LPIN1*	Nucleus, ER, cytosol	Phosphatidic acid hydrolysis, transcription
*LPIN2*	ER, cytosol	Phosphatidic acid hydrolysis
*LPIN3*	ER, cytosol	Phosphatidic acid hydrolysis
*MDH2*	Mitochondrial matrix	Tricarboxylic acid cycle, malate metabolic process
*PPARD*	Nucleus	Fatty acid beta-oxidation, transcription
*PPARG*	Nucleus	Induction of adipocyte differentiation, transcription
*PPARGC1A*	Nucleus	Fatty acid beta-oxidation, transcription factor
*PPARGC1B*	Nucleus	DNA-dependent regulation of transcription
*PRKAA1*	Cytosol	Fatty acid biosynthesis, signal transduction
*PRKAA2*	Cytosol	Fatty acid biosynthesis, signal transduction
*SCAP*	ER membrane, Golgi	SREBP target gene transcription activation
*SCD*	ER membrane	Fatty acid biosynthesis
*SLC27A1*	Plasma and ER membranes	Fatty acid transport
*SLC2A4*	Plasma membrane, cytosol	Glucose transport, glucose homeostasis
*SREBF1*	Golgi and ER membranes, nucleus	Transcription regulation
*THRSP*	Nucleus	Lipid metabolic process

^1^Entrez Gene, National Center for Biotechnology Information (NCBI)

## Results and discussion

### Animal performance

A primary objective was to manipulate the profile of ruminal fermentation products (e.g., increase propionate) through feeding diets with different levels of starch and fiber. Classical studies showed that intravenous infusions (14-day) of propionate or glucose (relative to saline, acetate, or lactate) into steers promoted greater in vitro incorporation of glucose, acetate, and lactate into subcutaneous adipose tissue lipid [18]. This response was accompanied by greater fatty acid synthetase (FASN), acetyl-CoA carboxylase-α (ACACA), and ATP-citrate lyase (ACLY), as well as unchanged malate dehydrogenase 2, NAD (mitochondrial) (MDH2) activity primarily when glucose was infused. The relative potency of substrates for inducing lipogenesis in ruminant adipose tissue was proposed to be glucose > propionate ≥ lactate > acetate [18].

Despite the well-defined greater rates of lipogenesis in subcutaneous adipose tissue of cattle [19], work also has shown greater intramuscular adipose FASN activity in 16–18 month-old steers fed high-starch (i.e., corn grain) vs. low-starch diets (i.e., corn silage) [20]. Intramuscular adipose tissue from steers fed the high-starch diet incorporated more glucose than acetate into glyceride-fatty acids and this response was augmented by age [20]. Due to gluconeogenesis in liver, both propionate and lactate can indirectly modulate adipose tissue lipogenesis through increased glucose synthesis, availability, or both. The glucogenic effect of high-starch diets is often accompanied by an enhanced insulin response [21], a well-known adipogenic/lipogenic signal in rodents [22]. There is some indication, however, that insulin in mature cattle is not as effective in increasing in vivo rates of lipogenesis in subcutaneous adipose as it is in rodents [23]. The responsiveness to insulin and abundance of insulin receptors increases dramatically as preadipocytes undergo terminal differentiation into adipocytes [7].

**Figure 1:**
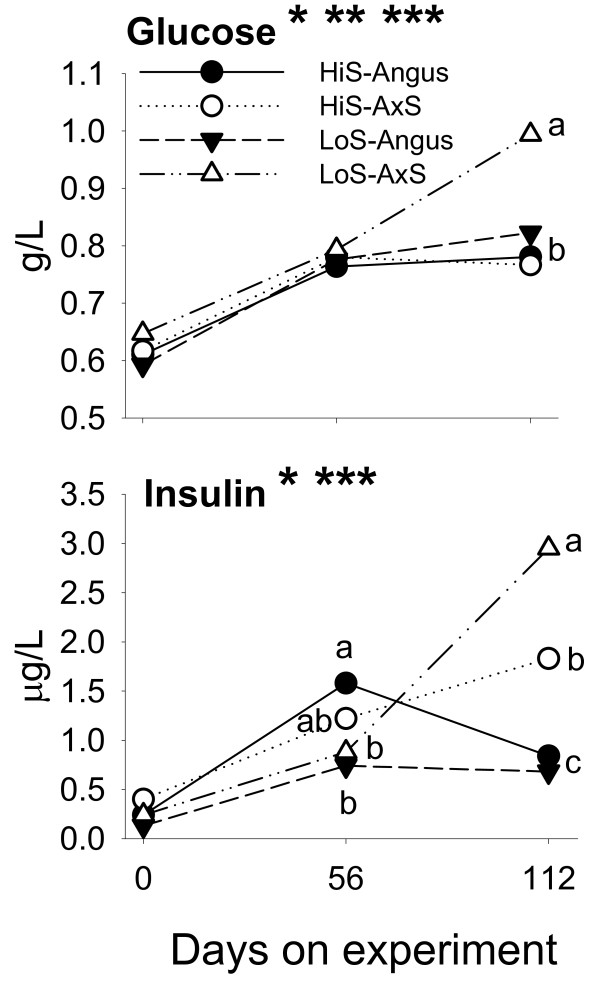
**Blood serum concentrations of glucose and insulin during the growing phase in Angus or A × S steers (n = 6/type) fed a high-starch (HiS, n = 3/type) or low-starch (LoS, n = 3/type) diet**. Asterisks denote (P < 0.05): *Time effect; **Diet effect; ***Steer type effect. Glucose had (P < 0.05) a diet × steer type interaction effect. Insulin had (P < 0.05) diet × steer type and diet × steer type × day interactions. Superscripts denote differences (P < 0.06) among treatments at specific time points.

All steers in our study consumed increasing amounts of feed as the study progressed (Additional File 1), a response associated with increasing concentrations of plasma hydroxybutyrate (**BHBA; **Additional File 1), glucose, and insulin between days 56 and 112 (Figure 1). Despite the greater energy content (ca. 20%) in the high-starch (**HiS**) vs. low-starch (**LoS**) diet, calculated energy intake did not differ significantly during the study (Table 2). However, it was evident that the greater feed intake by Angus × Simmental (**A × S**) steers fed LoS led to numerically greater amounts of energy consumption partly explaining greater blood glucose and insulin on day 112 (Figure 1). In general, the high-starch diet was most efficacious in terms of growth and performance during the first 56 days of the study regardless of steer type (Table 2). In addition, the high-starch diet resulted in numerically-greater feed efficiency and lower residual feed intake during the feeding period. Marbling score on day 112 was greater in Angus steers but was not affected by diet. Previous early-weaned studies with A × S steers showed that feeding high-starch diets containing ca. 35% to 60% more net energy for gain (**NE**_G_) than low-starch diets resulted in greater body weight, daily rates of gain, and greater feed efficiency despite similar rates of feed intake [17,24]. It is not surprising in our study that the overall diet effect (i.e., for the 112 day study) was non-significant on most performance measures because NE_G _intake due to HiS and LoS did not differ (Table 2; Additional File 1).

**Table 2:** Animal performance in response to a high-starch (HiS) or low-starch (LoS) diet during a 112-d growing phase.

	Treatments							
					
	HiS		LoS			*P *value		
			
Item	Angus	A × S	Angus	A × S	SEM	Diet	Steer type	Diet × Steer type
Body weight (kg)								
Birth	39.9	40.5	36.3	40.1	1.6	0.23	0.20	0.34
Initial, d 0	165	177	135	195	15	0.36	0.26	0.39
d 56	243	254	196	268	14	0.27	0.02	0.07
Final, d 112	331	352	294	373	16	0.61	0.01	0.10
ADG (kg/d)								
0 to 56 d	1.40	1.38	1.08	1.32	0.07	0.02	0.15	0.09
56 to 112 d	1.56	1.73	1.74	1.86	0.08	0.11	0.11	0.75
0 to 112 d	1.48	1.56	1.41	1.59	0.07	0.77	0.10	0.47
Dry matter intake (kg/day)								
0 to 56 d	6.34	6.24	5.88	7.79	0.42	0.23	0.06	0.04
56 to 112 d	7.45	7.42	8.04	9.56	0.52	0.03	0.18	0.17
0 to 112 d	6.73	6.75	6.77	8.56	0.45	0.07	0.04	0.05
NE_G _intake (Mcal/day)^1^								
0 to 56 d	9.07	8.93	6.99	9.27	0.56	0.16	0.09	0.06
56 to 112 d	10.6	10.6	9.57	11.4	0.67	0.81	0.21	0.21
0 to 112 d	9.77	9.67	8.13	10.2	0.57	0.36	0.12	0.09
Residual feed intake	-0.63	-1.58	1.22	1.10	0.62	0.006	0.41	0.52
Feed efficiency^2 ^(kg/kg)4								
0 to 56 d	0.22	0.22	0.18	0.17	0.02	0.04	0.68	0.75
56 to 112 d	0.21	0.24	0.22	0.19	0.01	0.29	0.86	0.14
0 to 112 d	0.22	0.23	0.21	0.18	0.01	0.09	0.77	0.35
Day 112 ultrasound								
Marbling score	4.37	3.97	4.90	4.05	0.24	0.21	0.06	0.39
Back fat (mm)	0.21	0.16	0.14	0.13	0.03	0.16	0.43	0.62
Muscle depth (mm)	53.4	51.8	53.0	48.9	3.3	0.61	0.52	0.74

^1 ^Estimated from actual dry matter intake (kg/day) × calculated NE_G _(1.19 or 1.43 Mcal/kg diet dry matter for HiS or LoS).

^2^ADG/feed intake.

Performance responses, including feed intake, energy intake, and residual feed intake from the subset of steers used for transcript profiling was comparable to the whole group of animals in the entire experiment (Additional File 1) suggesting that the steers randomly chosen for transcript profiling were representative of the entire group. Furthermore, each steer served as its own control because of the repeated sampling over time. Similar approaches for transcript profiling studies of skeletal muscle tissue have been used previously [8,11].

Previously, high-starch vs. low-starch diets fed to early-weaned A × S steers (n = 19–20/diet) for 100 days resulted in moderately greater intramuscular fat (3.6% vs. 3.2%) measured via ultrasound [17]. Our steers fed LoS, however, consumed greater overall amounts of energy than those fed LoS in the study of Schoonmaker et al. [17]. We were unable to obtain reliable measurements of marbling at 56 days of feeding but mRNA expression data discussed in the subsequent sections is indicative of diet effects on adipogenesis and lipid filling. Because the vast majority of metabolic enzymes in mammals are regulated at the transcriptional level [2], measurement of mRNA for multiple genes and their networks in a pathway should expand our understanding of muscle and fat development [7] in response to nutrition, genotype, and their interaction [9,11].

### Transcriptional regulatory networks of adipogenesis in LL

Several growth factors in blood such as insulin, insulin-like growth factor-1 (IGF-I), growth hormone, thyroid hormones (i.e., triiodothyronine), as well as vitamins [25], are required to stimulate proliferation of pre-adipocytes [7]. Classical studies in cattle demonstrated subcutaneous adipocyte hyperplasia between 4 and 7 months of age [26], and between 11 and 17 months of age [27]. There also are well-defined age-related increases in subcutaneous adipose ACLY, glucose-6-phosphate dehydrogenase (G6PD), ACACA, and FASN activity in growing crossbred steers fed high-starch vs. low-starch diets, which correlated with in vitro lactate and acetate incorporation into lipid [19,23]. During rapid post-weaning growth it is possible that a population of pre-adipocytes exits the proliferative phase and enters terminal differentiation [7].

### PPARG networks

*PPARG *role in marbling deposition could be crucial because in non-ruminants most pro-adipogenic factors seem to function at least in part by activating PPARγ expression or activity [22]. Up-regulation of PPARγ is sufficient to induce adipocyte differentiation in vitro and no factor has been discovered that promotes adipogenesis in the absence of PPARγ [22]. Terminal differentiation of adipocytes requires up-regulation of mRNA of fatty acid binding protein 4 (*FABP4*), *G6PD*, *FASN*, and *ACACA*, among others, which are under the control of PPARγ [7,28]. Additional PPARG-target genes [7,22] analyzed in the present study included: *ACLY*, CD36 molecule [thrombospondin receptor] (*CD36*), diacylglycerol O-acyltransferase homolog 1 and 2 (*DGAT1 *and *2*), stearoyl-CoA desaturase (*SCD*), mitochondrial glycerol-3-phosphate acyltransferase (*GPAM*), insulin induced gene 1 (*INSIG1*), insulin receptor *(INSR*), insulin receptor substrate 1 (*IRS1*), solute carrier family 2 [facilitated glucose transporter] member 4 (*SLC2A4*), solute carrier family 27 [fatty acid transporter] member 1 (*SLC27A1*), and sterol regulatory element binding transcription factor 1 (*SREBF1*).

**Figure 2:**
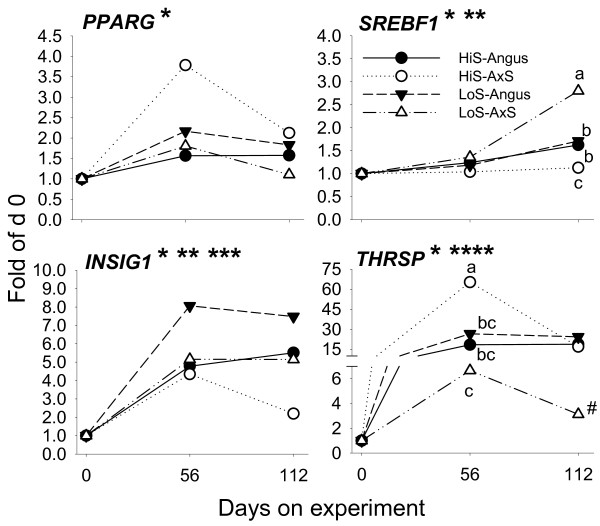
**mRNA expression patterns of genes associated with ligand-dependent transcriptional control of adipocyte differentiation and ligand-dependent activation of lipogenesis (*PPARG*), and transcriptional regulation of lipogenesis (*THRSP*, *SREBF1*, *INSIG1*) in Angus or A × S steers (n = 6/type) fed a high-starch (HiS, n = 3/type) or low-starch (LoS, n = 3/type) diet**. Fold-change expression during the growing phase is expressed relative to day 0. Pooled SEM: *PPARG*, 0.5; *THRSP*, 8.8; *INSIG1*, 0.9; *SREBF1*, 0.2. Asterisks denote (P < 0.05): *Time effect; **Diet effect; ***Steer type effect; ****Tendency (P = 0.13) for diet effect. *SREBF1 *and *THRSP *had significant (P < 0.05) diet × steer type and diet × steer type × day interactions. Superscripts denote differences (P < 0.05) among treatments at specific time points. #P = 0.10, LoS-A × S vs. LoS-Angus, HiS-Angus, HiS-A × S.

**Figure 3:**
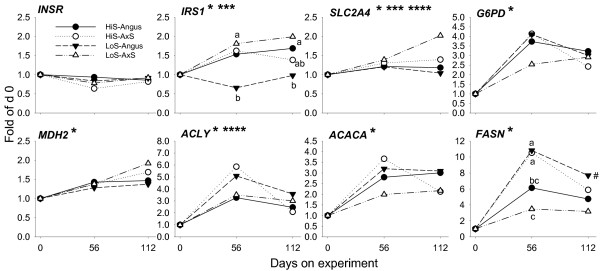
**mRNA expression patterns of genes associated with insulin signaling (*INSR*, *IRS1*), insulin-stimulated glucose uptake (*SLC2A4*), cytosolic NADPH generation (*G6PD*), malate-aspartate shuttle (*MDH2*), cytosolic acetyl-CoA synthesis from citrate (*ACLY*), and de novo fatty acid synthesis (*ACACA*, *FASN*) in Angus or A × S steers (n = 6/type) fed a high-starch (HiS, n = 3/type) or low-starch (LoS, n = 3/type) diet**. Fold-change expression during the growing phase is expressed relative to day 0. Pooled SEM: *INSR*, 0.1; *IRS1*, 0.2; *SLC2A4*, 0.2; *G6PD*, 0.6; *MDH2*, 0.2; *ACLY*, 0.7; *ACACA*, 0.5; *FASN*, 1.2. Asterisks denote (P < 0.05): *Time effect; **Diet effect; ***Steer type effect; ****Tendency (P < 0.13) for diet × steer type × day interaction. *IRS1 *and *FASN *had (P < 0.05) a diet × steer type interaction. *FASN *had (P < 0.05) a diet × steer type × day interaction. Superscripts denote differences (P < 0.05) among treatments at specific time points.

**Figure 4:**
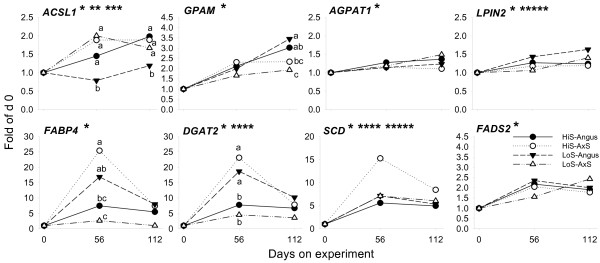
**mRNA expression patterns of genes associated with intracellular activation of fatty acids to acyl-CoA (*ACSL1*), glycerol-3-phosphate acylation (*GPAM*), acylglycerol-phosphate acylation (*AGPAT1*), diacylglycerol formation (*LPIN2*), intracellular fatty acid transport (*FABP4*), acylation of diacylglycerol and formation of TAG (*DGAT2*), and desaturation (*SCD*, *FADS2*) in Angus or A × S steers (n = 6/type) fed a high-starch (HiS, n = 3/type) or low-starch (LoS, n = 3/type) diet**. Fold-change expression during the growing phase is expressed relative to day 0. Pooled SEM: *ACSL1*, 0.2; *GPAM*, 0.3; *AGPAT1*, 0.1; *LPIN2*, 0.1; *FABP4*, 4.2; *DGAT2*, 3.8; *SCD*, 1.8; *FADS2*, 0.2. Asterisks denote (P < 0.05): *Time effect; **Diet effect; ***Steer type effect; ****Tendency (P = 0.08–0.11) for diet × steer type × day interaction; *****Tendency (P = 0.11–0.13) for steer type effect. *ACSL1 *and *GPAM *had (P < 0.05) diet × steer type × day interactions. *FABP4 *and *DGAT2 *had (P < 0.05) diet × steer type interactions. Superscripts denote differences (P < 0.05) among treatments at specific time points.

Recent in vitro studies showed that bovine perimuscular pre-adipocytes induced to differentiate with insulin and glucocorticoids had greater mRNA expression of *PPARG*, *SREBF1*, *FABP4*, acyl-CoA synthetase long-chain family member 1 (*ACSL1*), and *FASN *after 2 days in culture compared with control [29]. Further, *PPARG *and *FABP4 *expression remained elevated through 8 days in culture, suggesting both were essential to sustain the differentiation program or that they are abundantly expressed in mature adipocytes [29]. In our study, overall expression of *PPARG *mRNA increased ca. 2-fold by day 56 primarily due to the large increase in A × S steers fed HiS. Expression of several of its potential target genes (e.g., *ACACA*, *FASN*, *FABP4*, *SCD*) increased to a much greater extent (Figure 2, 3, 4). Similar responses were observed recently in high-marbling Wagyu × Hereford vs. low-marbling Piedmontese × Hereford heifers at 7 months of age relative to 3 months of age [11].

Among all potential PPARγ target genes, we found a highly-positive and significant correlation between *FABP4 *and *PPARG *regardless of steer type or diet (Additional File 2) which is suggestive of an increase in PPARγ activity. We recently presented direct evidence, via use of the PPARγ agonist rosiglitazone, that *SREBF1*, *FASN*, *ACACA *and potentially *FABP4 *and thyroid hormone responsive (SPOT14 homolog, rat) (*THRSP*) are *PPARG *target genes in the bovine [30]. Some of the above genes remained elevated through day 112 regardless of diet or steer type. Along with *PPARG*, the expression patterns of *FASN*, *FABP4*, *SCD*, *ACLY*, *THRSP*, and *DGAT2 *clustered together into the most up-regulated group of genes (Additional File 1). This observation together with the correlation data (Additional File 2) for *FASN*, *SCD*, *ACLY*, and *DGAT2 *with *PPARG*, in particular, provides additional evidence that they likely are *PPARG *downstream genes in cattle. Considering the essential role of PPARγ in adipogenesis [7], we speculate that these genes could be considered an essential subset for adipocyte differentiation and lipid filling. Previous work showed intramuscular adipocyte hyperplasia (i.e., proliferation) occurring at 11 through 17 months of age [27]. Increased adipocyte number in our study encompassing steers ca. 4–8 months of age is supported by some of the measured genes which are highly abundant in adipose compared with other tissues in mammals (e.g. *PPARG*, *DGAT2*, *FABP4*, *SCD*; [7]), and also by the increase in total fatty acid concentration in muscle tissue over time (Additional File 1).

We observed interaction effects (including tendencies) on the mRNA expression patterns of *FABP4*, *ACLY*, *FASN*, *SCD, GPAM*, and *DGAT2*. Hierarchical clustering analysis clearly highlighted differences in expression of these genes across steer type and diet combinations (Additional File 1). All these genes had the opposite response between steer types when fed the two diets particularly on day 56, e.g., feeding HiS to A × S steers resulted in the greatest response compared with A × S steers fed LoS. Also, Angus steers responded with greater values for most of these genes when LoS was fed (Figures 3, 4). There was a clear time effect on all the lipogenic genes (e.g., *ACACA*, *FASN*, *SCD*) with peak expression at 56 days and a moderate decrease thereafter. At 112 days, few of these genes had significant differences between groups, with an overall tendency for Angus steers to have greater values. In general, these data agree with marbling scores measured at 112 days showing greater responses in Angus steers.

The common trend for decreased mRNA expression after 56 days seems to suggest that the initial adipogenic response through 56 days did not influence intramuscular fat deposition by 112 days. There is evidence of positive correlations between mRNA expression of certain lipogenic genes/transcription regulators and intramuscular fat content in crossbred cattle [11]. However, it was intriguing that A × S steers fed HiS, with numerically lower marbling score at day 112, had the same pattern of expression among all adipogenic genes compared with Angus steers fed LoS, which in the end had numerically greater marbling scores. It also is noteworthy that A × S steers fed LoS had lower temporal expression of most genes driving adipogenesis (Figure 2, 3, 4) without discernable effects on marbling scores. Potential carry-over effects of high-starch diets on intramuscular fat content need to be evaluated during the finishing phase and/or at slaughter.

### SREBF1 networks

SREBP1 is a pro-adipogenic factor regulating transcriptional cascades primarily in rodent liver [4]. The transcription factor binds specific DNA domains [4] eliciting transcription of genes involved in lipid and cholesterol metabolism. A direct crosstalk with PPARγ or via generation of a lipid PPARγ-ligand whose identity is still unknown has been proposed as an additional mechanism during adipocyte differentiation [31]. We have evidence that SREBF1 is a PPARγ-target gene in bovine cells [30]. The role of SREBP1c on lipid homeostasis in rodent cells/tissues (primarily hepatocytes) has been well-established [7,32]. At normal levels of expression in murine liver, SREBP1c activates expression of *ACLY*, *G6PD*, *ACACA*, *FASN*, *SCD*, and *GPAM *leading to synthesis of palmitic acid, oleic acid, and formation of triacylglycerol (**TAG**) [32]. Similar responses have been reported in murine adipose tissue [33]. Recent data, however, casts some doubt on the essentiality of SREBP1 for regulation of rodent adipocyte lipogenic gene expression [34].

In our study, mRNA of *SREBF1 *was significantly greater between 56 and 112 days in all groups except A × S steers fed HiS (Figure 2). mRNA of *INSIG1 *had greater up-regulation by 56 days and remained elevated through 112 days in all groups except A × S steers fed HiS. The overall increase in expression of *SREBF1*, at least through the second portion of the feeding phase, is additional indication of an enrichment [22] of intramuscular adipocytes. Induction of adipogenesis in bovine bone marrow stromal cells led to greater *SREBF1 *mRNA several days after they were induced to differentiate [35]. It could be possible that changes in *SREBF1 *expression exert some level of control over intramuscular lipogenesis in growing cattle, but the substantial and sustained increase in expression of *INSIG1 *along with its high mRNA abundance relative to *SREBF1 *(Additional File 1) are indicative of low activity of SREBP1. Furthermore, a recent study in rodents found that lipogenic gene expression in adipose tissue was independent of SREBP1 [34]. Together, these observations cast doubt on the essentiality of this transcription factor in cattle intramuscular fat accumulation.

*INSIG1 *mRNA in murine adipose tissue is induced by activation of PPARγ but it occurs relatively late in the adipogenic program, preceded by peak of *PPARG *and *SREBF1 *mRNA expression [36]. In fact, both *PPARG *and *SREBF1 *activate transcription of *INSIG1 *and regulate its expression during adipogenesis [36]. The primary function of INSIG1 in adipose tissue or liver is to block processing of SREBP1 [33]. However, it is believed that concomitant increase in the activation of INSIG1 and SREBP1 is a counterbalance mechanism ("brake") to prevent uncontrolled SREBP1 action, i.e. lipogenesis [33,37]. Up-regulation of *INSIG1 *transcription in adipocytes effectively down-regulated expression of *PPARG *and *SREBF1*, essentially blocking differentiation of pre-adipocytes [33]. Thus, an increase in expression of *INSIG1 *provides a feedback signaling mechanism to restrict both lipogenesis and adipogenesis. Our data, however, do not seem to support such a mechanism in bovine muscle because the response in *INSIG1 *expression was greater in Angus steers, which had greater marbling scores at 112 days. In addition, Angus steers fed LoS had the greatest increase in expression of *INSIG1 *as well as numerically highest marbling scores and total fatty acids in muscle tissue (Additional File 1). *INSIG1 *is a PPARγ target gene in the mouse [36] and previous data from our laboratory provided evidence of the same in the bovine [30]. These findings point to a crucial role for PPARγ in controlling intramuscular lipogenesis in cattle. The pattern of *INSIG1 *expression together with its apparent regulation via PPARγ suggest that this gene might be a pro-lipogenic rather than anti-lipogenic factor as inferred in the mouse [36].

### THRSP networks

*THRSP *is another important gene during adipocyte differentiation in rodents and is partly regulated by thyroid hormone [28,38,39]. In rodents, thyroid hormone exerts sustained up-regulation of *THRSP *expression [28,38,39] and can by itself increase rates of lipogenesis in rodent adipose tissue. Both insulin and thyroid hormone can act synergistically in promoting the overall process of adipogenesis in rodents [40]. In pigs, however, it is believed that thyroid hormone acts synergistically with other growth factors such as insulin-like growth factor-I to promote adipocyte differentiation [7]. A microarray study previously identified *THRSP*, in addition to *SCD*, *FABP4*, and *SREBF1*, as one marker gene preferentially expressed in LL of Japanese Black vs. Holstein steers [41]. The former are widely-known to possess extremely high capacity for marbling. More recently, *THRSP *mRNA in LL was highly correlated (0.94) with intramuscular fat content in high-marbling Wagyu × Hereford but not in low-marbling Piedmontese × Hereford heifers [11]. Both thyroid hormone and insulin are positively and significantly correlated with feed intake, body weight, and average daily gain in cattle [42]. Insulin and high-carbohydrate diets induced *THRSP *mRNA in rodent liver [38] and adipose tissue [39].

Among novel results, we observed dramatic up-regulation by day 56 of *THRSP *transcription (Figure 2). A recent study reported marked *THRSP *in LL of crossbred Wagyu × Hereford vs. Piedmontese × Hereford heifers but at 25 to 30 months of age relative to 3 months of age [11]. The former had greater (10.7% vs. 5.3%) carcass intramuscular fat at slaughter. In our study, the increase in *THRSP *was not affected by dietary starch in Angus steers while in A × S steers fed HiS it resulted in a tremendous up-regulation of its mRNA abundance. As observed for classical adipogenic genes, A × S steers fed LoS had greater *THRSP *expression by 56 days but the magnitude of increase was lower compared with other groups. By 112 days, *THRSP *expression was only numerically greater compared to the pre-experimental value. In other groups, mRNA abundance of *THRSP *was maintained at >15-fold through day 112 compared with day 0. Insulin and thyroid hormone act synergistically to enhance THRSP-driven lipogenesis in rodent adipose, a mechanism that seems to be in line with the well-defined parallel increases in blood insulin and thyroid hormone during rapid growth in cattle [21,42]. We found positive correlations between both insulin and glucose with *THRSP *expression but only in Angus cattle regardless of diet (Additional File 2). For the most part, correlations between mRNA abundance of *THRSP *and expression of lipogenic genes and *PPARG *were positive and significant regardless of diet or steer type (Additional File 2). More detailed studies will have to be conducted in the future to determine the role, if any, of thyroid hormone alone or in combination with other hormones or growth factors in the process of adipogenesis during rapid cattle growth.

Despite the well-defined effect of thyroid hormone on rodent lipogenesis it also down-regulates *SREBF1 *mRNA expression, both in mouse liver [43] and human adipose tissue [44]. This effect might partly explain the contrasting expression patterns of *SREBF1 *and *THRSP *in A × S steers (Figure 2). Sustained up-regulation in expression of *THRSP *in LL of growing cattle would partly overcome the need for *SREBF1 *expression and activation of lipogenesis because *ACACA *and *THRSP*, at least in murine liver, are co-expressed [38] thus, *THRSP *regulation of *ACACA *in muscle cannot be discounted.

### Insulin signaling in LL inferred by gene expression, blood metabolites, and insulin

Short-term in vitro studies with bovine myogenic cells have demonstrated greater *INSR *and *IRS1 *protein, marked phosphorylation of IRS1, as well as greater protein of downstream effectors (phosphoinositide-3-kinase,SLC2A4) within minutes of insulin stimulation [45,46]. Insulin effectively stimulates muscle glucose oxidation and adipogenesis [22,47], partly through up-regulation of *IRS1 *transcription [48] and through activation of downstream signaling cascades including transcription factors (*SREBF1*), nuclear receptors (e.g., *PPARG*, *PPARGC*), and their gene targets (e.g., *FABP4*, *FASN*).

Among *INSR *and *IRS1*, only the latter had an overall temporal increase in expression, with a pattern similar in all groups except Angus steers fed LoS, which had a decrease in expression throughout the study (Figure 3). The greater temporal expression pattern of SLC2A4was significant only for A × S steers fed LoS. Both, *IRS1 *(~7% of total genes; Additional File 1) and *SLC2A4 *(~8% of total genes) were among the most abundant genes measured in our experiment, suggesting an important role in LL. Among isoforms of *IRS*, expression of *IRS1 *appears essential for the induction of adipogenesis mediated by insulin in rodents [22]. The pattern of expression of *IRS1 *in our experiment does not support such a role for this gene in cattle LL. Angus steers fed LoS, which at 112 days had numerically greater marbling score (Table 2) and total fatty acids in muscle tissue (Additional File 1), had the lowest increase in expression of *IRS1 *and lowest plasma insulin. The low insulin together with low glucose in LoS-fed Angus indicates that they were more insulin sensitive. The opposite was observed for A × S steers fed LoS, which had the lowest numerical marbling score (Table 2) and lowest increase in expression of lipogenic genes despite having the largest increase in expression of *IRS1*. These results seem to cast doubt on an essential role of *IRS1 *in adipogenesis/lipogenesis.

The importance of adipose PPARγ to maintain systemic (muscle and adipose) insulin sensitivity in non-ruminants has been convincingly demonstrated [6]. Blood insulin and glucose were clearly greater in A × S steers fed LoS, particularly during the second half of the growing phase (Figure 1), which could be taken as an indication of reduced insulin sensitivity. These cattle were unique because they consumed the greatest amounts of dry matter and energy but their growth and feed conversion efficiency rates did not differ substantially from other animals (Table 2; Additional File 1). Clustering analysis of gene expression patterns underscored the marked differences in mRNA expression induced by feeding a low-starch diet to these animals (Additional File 1). Insulin resistance in A × S steers fed LoS also is supported by the lower temporal response of *PPARG*-target genes (e.g., *ACACA*, *FABP4*) pointing to lower activity of PPARγ, which could be caused by reduced insulin signaling.

The above data are intriguing and, to some extent, counterintuitive. Low-starch diets should not reduce insulin sensitivity as suggested by data from Angus steers in which feeding LoS led to lower plasma insulin. However, results with A × S steers fed LoS agree with a previous study showing that intramuscular fat tissue from steers fed a low- vs. high-starch diet was less sensitive to insulin-stimulated glucose use for lipogenesis [49]. An insulin-resistant state in A × S steers fed LoS can partly help explain the lack of response in expression of adipogenic genes *ACACA*, *FASN*, *FABP4*, *GPAM*, and *DGAT2 *throughout the study (Figure 3, 4; Additional File 1) despite greater overall fold-change in *SREBF1 *mRNA at 112 days (Figure 2; Additional File 1). Transcriptional regulation of *SREBF1 *in most non-ruminant models is sensitive to insulin, which under times of carbohydrate excess leads to stimulation of fatty acid synthesis and TAG deposition both in adipose [31] and liver [32]. The decrease in insulin sensitivity also may explain the lower expression of *THRSP *in A × S steers fed LoS, because insulin is essential for its full expression [40]. Overall, our data suggest that low-starch diets induced insulin resistance in LL of A × S steers and highlights a difference in response to diet between pure bred and crossbred cattle. Further research with greater number of cattle seems warranted to examine more directly insulin sensitivity in LL under these or similar nutritional management schemes.

### Intracellular LL tissue energy metabolism

#### Nuclear receptors and co-activators involved in energy metabolism

During rapid muscle growth the energetic costs associated with protein deposition and intramuscular lipogenesis in LL tissue are expected to increase, as shown by enzymatic activity measurements over time [9,19,23]. *PPARD *is a poorly-studied nuclear receptor with a potentially important role in skeletal muscle energy metabolism. *PPARD *mRNA is several fold more abundant than PPARα in murine skeletal muscle and effectively performs the same function, i.e. activation of fatty acid oxidation [50]. The co-activators peroxisome proliferator activated receptor coactivator-1α (*PPARGC1A*) and -1β (*PPARGC1B*) also are involved in stimulating oxidative metabolism in skeletal muscle [51]. Only Angus steers fed LoS had a significant increase in transcript abundance of *PPARD *(Additional File 1). *PPARGC1A *was characterized by a blunted temporal response in all groups except Angus steers fed HiS, which had a large increase by 112 days (Additional File 1). *PPARGC1B *was consistently up-regulated in steers fed HiS (Additional File 1). We found a positive correlation (except in Angus steers fed LoS) between both *PPARGC1A *and *PPARGC1B *with *ACSL1*, encoding a protein showing to channel fatty acids towards oxidation in heart muscle tissue [52]. *ACSL1 *had a significant increase in expression over time in all groups except Angus steers fed LoS (Figure 4).

The role of *ACSL1 *in fatty acid oxidation is likely confounded in our study because in adipose tissue this protein channels fatty acids toward synthesis of TAG, while in muscle it channels fatty acid toward oxidation [53]. The core biopsy tissue in our experiment contained both adipocytes and muscle cells. However, the blunted response in expression of this gene in Angus fed LoS, with greater marbling score, are suggestive of a more prominent role for ACSL1 in channeling fatty acids toward oxidation in cattle muscle cells. This is supported by the positive correlation between ACSL1 with *PPARGC *isoforms [51].

Activation of PPARD in vitro has been associated with increased fatty acid oxidation through an AMPK-dependent mechanism [54]. AMPK is a heterodymeric enzyme that functions as an intracellular "fuel gauge" that monitors changes in energy status, and it is activated upon an increase in AMP/ATP ratio essentially reducing anabolic pathways such as lipogenesis [55]. The catalytic subunits of AMPK are encoded by protein kinase, AMP-activated, alpha 1 and 2 catalytic subunits (*PRKAA1 *and *PRKAA2*). These two genes did not respond to dietary starch (Additional File 1). Interestingly, *PRKAA2 *expression within each steer type had the opposite pattern compared with *PPARD*. Furthermore, *PRKAA2 *expression during the study increased only in A × S steers but not in Angus.

Angus steers fed LoS had a greater increase in expression of *PPARD *and numerically greater increase in expression of *PRKAA1 *compared with feeding HiS (Additional File 1). This might suggest that feeding LoS resulted in greater utilization of preformed fatty acids for oxidation through greater expression of *PRKAA1*. Increasing fatty acid oxidation would be a means to spare glucose for de novo fatty acid synthesis in intramuscular adipose tissue. This suggestion is supported by the temporal expression of *ACLY *in Angus steers fed LoS. In fact, this group had greater increase in expression of *FASN *by 56 days and greater concentrations of short-chain fatty acids in LL tissue (Additional File 1). This mechanism in Angus steers fed LoS is supported only by *PPARD *data, while remaining genes potentially involved in fatty acid oxidation (e.g., *PPARGC1A *and *B*, *ACSL1*) seem to indicate lower oxidation of fatty acids. A factor that confounds interpretation, as indicated above, is the different composition of cells (i.e., adipocytes, muscle cells) in the core biopsy tissue. Greater expression of *ACSL1 *is sometimes associated with insulin resistance due to direct effects of long-chain fatty acyl-CoA [56]. An increase in the intracellular pool of long-chain fatty acyl-CoA in A × S steers fed LoS is supported by the sustained up-regulation in *CD36 *(discussed below; Additional File 1) and up-regulation of *SLC27A1 *by day 112. Both proteins are involved in uptake of preformed long-chain fatty acids [57].

#### Intracellular energy sensors and insulin action

A hallmark of normal tissue insulin signaling is the enhanced uptake of long-chain fatty acids through both passive diffusion as well as protein-mediated transport [57]. The fatty acid translocase FAT/CD36 plays a major role in this process [57]. Both, contraction and insulin appear to up-regulate muscle CD36 recruitment from intracellular stores to the plasma membrane at least in part through the action of AMPK [58]. Longitudinal mRNA expression and mRNA abundance of *CD36 *and *PRKAA2 *were greater in A × S steers regardless of diet, and essentially followed the opposite pattern relative to Angus steers (Additional File 1). Both genes exhibited the clearest effect of steer type on transcript expression among the 31 genes examined and their expression clustered together (Additional File 1). Studies have shown that null mutation of *CD36 *in mouse skeletal muscle leads to impaired AMPK-stimulated (measured via PRKAA2 phosphorylation state) fatty acid oxidation in oxidative fibers [59]. Those previous results would imply that lower *CD36 *and *PRKAA2 *mRNA expression in Angus steers potentially serves as a mechanism to divert more long-chain fatty acids taken up via *SLC27A1 *(greater in Angus steers; Additional File 1) towards TAG synthesis.

It could be possible that *CD36 *and *PRKAA2 *work in concert with *LPIN *isoforms or as yet unidentified nuclear receptors to regulate cellular fatty acid oxidation [60]. The lipin 1 isoform (*LPIN1*) was shown to selectively activate a subset of *PPARGC1A*-target pathways, including fatty acid oxidation and mitochondrial oxidative phosphorylation in murine liver [61]. In our data, *LPIN2 *expression was positively correlated with insulin in both Angus and A × S steers fed HiS, suggesting that it might be associated with adipogenesis.

## Conclusion

**Figure 5:**
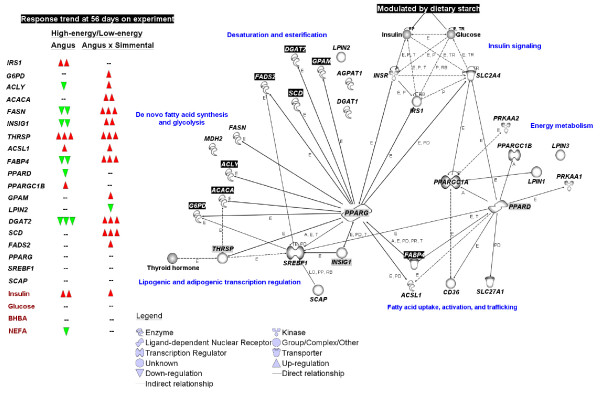
**Major trends in mRNA expression at 56 days on experimental diets and summary network analysis among genes**. The complete network including currently-known relationships among genes in non-ruminants from the Ingenuity Pathway Analysis^® ^knowledge base is available in Additional File 1. The *PPARG *relationships depicted are based on the relative responses found in the present study and do not represent actual fold-changes in expression as those are depicted in Figure 2–4. Relationships dealing with insulin and glucose signaling via *INSR*, *SLC2A4*, *IRS1*, and their link with *PPARG *were from the Ingenuity Pathway Analysis^® ^knowledge base and have been discussed to some extent in the review by Fernyhough et al. [6]. Other relationships from the Ingenuity Pathway Analysis^® ^knowledge base include those encompassing genes associated primarily with energy metabolism. Genes with black-colored background had fold-changes in mRNA expression ≥ 2-fold in at least one time point in all groups. Genes with grey-colored background appear central for transcriptional regulation of adipogenesis and energy metabolism. Together with clustering analysis (Additional File 1), data suggest that PPARγ activity through up-regulation of *FABP4*, *DGAT2*, *FASN*, and *SCD *is crucial for adipogenesis. The transcription factor *THRSP *was dramatically up-regulated by the high-energy diet regardless of steer type and might constitute an important transcription regulator of adipogenesis.

Results involving gene markers of mature adipocytes (e.g., *PPARG*, *THRSP*, *SCD*) provided evidence of intramuscular adipose tissue differentiation during the early portion of the growing phase. Although ultrasound evaluation of intramuscular fat did not detect differences due to diet at the end of the study, gene expression patterns suggest that dietary starch level might alter pathways associated with intramuscular adipose tissue development. This was most evident in A × S steers fed high-starch vs. low-starch, which suggests that cattle genetics also might be an important factor to consider when developing management strategies to manipulate skeletal muscle composition. The resulting gene networks (Figure 5) underscored a central role for *PPARG *in controlling transcription of genes which are known to coordinately regulate adipocyte differentiation and lipid filling in non-ruminants partly via insulin. Analysis also highlighted a putative role of *PPARD*, in coordination with *PPARGC1A *and *PPARGC1B*, in controlling intracellular energy metabolism. Unlike non-ruminants, *INSIG1 *rather than *SREBF1* appears to play a more important role in cattle muscle adipogenesis. We propose that a network of transcription regulators and nuclear receptors including *PPARG*-target genes,* INSIG1*, and *THRSP*, coordinate activation of adipocyte differentiation and lipid synthesis.

More functional studies are clearly needed to determine whether up-regulation in expression of transcription factors and nuclear receptors via diet at an early age can induce precocious adipocyte differentiation and ultimately determine intramuscular fat deposition in the carcass. There will be a need for dissecting intramuscular adipose and muscle tissue so that cell-specific analysis can be made. Other limitations of the study were the lack of additional functional data (e.g., measurement of oxidation or TAG synthesis), and actual carcass data. Despite those limitations, our data provided novel insights into *longissimus lumborum *energy sensing and lipogenic gene networks as affected by dietary starch level and genotype.

## Methods

### Experimental animals and management, diets, and sampling

The study utilized a subset of 12 animals selected from a larger study encompassing 29 early-weaned (134 ± 10 day age at weaning) purebred Angus (n = 17) and Angus × Simmental (n = 12) steers from the University of Illinois beef cattle herd. After a 3-week adjustment period in which all steers were fed the same corn silage-based diet (850 g/kg corn silage and 150 g/kg wet distiller's grains, as-fed basis), 6 Angus and 6 A × S steers from the larger groups that were fed a high-starch (1.43 Mcal/kg diet dry matter; n = 3/steer type) or low-starch (1.19 Mcal/kg diet dry matter; n = 3/steer type) diet for 112 days after weaning (i.e., growing phase) were chosen randomly for LL biopsies. Dietary treatments in this study were specifically designed to provide contrasting levels of starch and fiber while providing sufficient energy, i.e. calculated net energy of gain in diets HiS and LoS differed by ca. 20% but was adequate in both cases to support ≥ 1.5 kg body weight per day [62]. Both diets were formulated to be isonitrogenous. The low-starch/high-fiber diet contained (g/kg dry matter) 350 corn silage, 200 corn gluten feed, 380 soyhulls, 30 cracked corn, and 30 soybean meal (490 g/kg crude protein). The high-starch/low-fiber diet contained (g/kg dry matter) 200 corn silage, 680 cracked corn, and 110 soybean meal (490 g/kg crude protein). Both diets contained (g/kg dry matter) 10 limestone/dicalcium phosphate/mineral/vitamin/urea/dry molasses mixture. Calculated dietary fiber content was 5.9% with HiS and 24% with LoS. Calculated NE_G _for the low-starch/high-fiber diet was 1.19 Mcal/kg dry matter and 1.43 Mcal/kg dry matter for the high-starch/low-fiber diet. All diets were offered on an ad libitum basis. Steers had an individual electronic identification ear tag, and individual feed intake data were collected using the GrowSafe^® ^system (GrowSafe Systems Ltd., Alberta, Canada). Residual feed intake was calculated by regression [63] of actual dry matter intake against average metabolic body weight (body weight^0.75^) and average daily gain (**ADG**).

Steer weights were recorded on consecutive days before, 56, and 112 days after the start of treatments (i.e., 155 ± 10 day age). Individual-animal ADG and daily dry matter intake were used to estimate feed conversion efficiency (gain/feed, kg/kg; Table 2). Ultrasound images of LL area were captured at 112 days of the growing phase using a 500 V Aloka (Corometrics Medical Systems, Inc., Wallingford, CT) ultrasound with a 3.5-MHz transducer fitted to a custom beef animal standoff. Data were analyzed with AUSkey System Software (Animal Ultrasound Services, Ithaca, NY). Commercial vegetable oil was applied to the site of measurement to decrease sound wave attenuation associated with hair coat.

Blood serum metabolites were analyzed following standard protocols at the Veterinary Diagnostics Laboratory, College of Veterinary Medicine, University of Illinois. Serum insulin concentration was quantified using a commercial bovine insulin ELISA kit (cat# 10-1201-01, Mercodia AB, Uppsala, Sweden).

Muscle biopsies were collected at 0, 56, and 112 days relative to the start of feeding treatment diets (i.e., 155 ± 10 day age) under a protocol (#05095) approved by the University of Illinois Animal Care and Use Committee. Specific details of the biopsy procedures can be found in Additional file 1.

### RNA extraction, RNA quality assessment, real time quantitative PCR (qPCR), primer design and evaluation, sequencing, internal control gene (ICG) evaluation, and muscle tissue fatty acid analysis

Specific details of these procedures are presented in Additional File 1. Special attention was given to the selection and evaluation of ICG for normalization of qPCR data. Briefly, microarray data from LL muscle [64] were mined to select potential ICG (Additional File 1) using established protocols from our laboratories [65-67]. Genes selected from the microarray data which had a stable expression ratio (i.e., 1.0 ± 0.2; sample/reference) included arrestin domain containing 1 (*ARRDC1*), endothelial differentiation, sphingolipid G-protein-coupled receptor, 1 (*EDG1*), chromosome 20 open reading frame 196 (*C20ORF196*), single stranded interacting protein 2 (*RBMS2*), and mitochondrial GTPase 1 homolog (*MTG1*). Previously-used [8,68,69] genes for normalization of cattle skeletal muscle tissue include actin beta (*ACTB*), glycerol-3-phosphate dehydrogenase (*GAPDH*), and cyclophilin (*PPIA*, *PPIB*). These transcripts were highly unstable (Additional File 1) and would have been rejected from subsequent analysis based on our initial criteria above. To the 5 genes were included ribosomal protein S15a (*RPS15A*) and ubiquitously-expressed transcript (*UXT*), which were previously identified as suitable ICG in bovine mammary tissue [67], and also *ACTB *and *GAPDH*. Absence of co-regulation among these selected genes was evaluated through Ingenuity Pathway Analysis^® ^(Additional File 1). Expression stability was evaluated using geNorm software  following the procedures of Vandesompele et al. [70] described in Additional File 1. A similar approach was used recently with gene expression data from cattle muscle [71]. Genes selected as ICG based on absence of co-regulation (Additional File 1) and geNorm analysis (Additional File 1) included *RBMS2*, *RPS15A*, *UXT*, and *MTG1*. The geometric mean of these 4 genes was used to normalize gene expression data in the present study.

### Statistical analysis

Growth performance, blood metabolites and insulin, LL fatty acid concentration, and qPCR data were analyzed as a factorial experiment, with diet and time as the two factors, using the MIXED procedure in SAS (SAS Institute) with repeated measures [72]. Prior to statistical analysis, normalized qPCR data were transformed [73,74] to fold-change relative to day 0 (i.e., before animals were started on experimental diets). To estimate standard errors at day 0 and prevent biases in statistical analysis, normalized qPCR data were transformed to obtain a perfect mean of 1.0 at day 0, leaving the proportional difference between the biological replicate. The same proportional change was calculated at all other time points to obtain a fold-change relative to day 0. Fixed effects in the statistical model for each variable analyzed (i.e., genes, blood metabolites, performance) included diet, steer type, days on experiment, diet × steer type, diet × days on experiment, steer type × days on experiment, and diet × steer type × days on experiment. Random effect was steer within diet. An autoregressive covariate structure was used [72]. All means were compared using the PDIFF statement of SAS. Significance was declared at *P *≤ 0.06. Pearson correlations among genes and performance variables within steer type and diet combination were obtained using the CORR procedure in SAS (Additional File 2).

### Clustering analysis

Hierarchical (Additional File 1) and k-means (Additional File 1) clustering was performed using fold-changes in mRNA expression for each steer type and diet combination on day 56 and day 112 relative to day 0 using Genesis software [75].

### Gene network analysis

Summary networks among genes (Figure 5; Additional File 1) were developed using the web-based software package Ingenuity Pathway Analysis^® ^(; Redwood City, CA). The networks were generated using the respective gene identifiers and not the actual fold-changes in expression which are already depicted in Figure 2, 3, and 4. Connections among genes were based on known relationships available in the Ingenuity Pathway Analysis^® ^knowledge based. This is a proprietary manually-curated database containing relationships from the published literature in rodents and humans.

## Abbreviations

ADG: average daily gain; A × S: Angus × Simmental steers; BHBA: hydroxybutyrate; LL: Longissimus lumborum; qPCR: real-time quantitative PCR; TAG: triacylglycerol(s).

## Authors' contributions

DEG, PP, and MB collected skeletal muscle biopsies and blood, performed RNA extraction, selection of internal control genes for qPCR, qPCR analysis, and data transformation. LLB, DBF, and JJL conceived, designed, and participated in the coordination of all aspects of the study. DEG, MB, and JJL drafted the manuscript. All authors read and approved the final manuscript.

## Supplementary Material

Additional File 1 **The file contains additional materials and methods** (biopsy procedure; RNA extraction, PCR, and primer design and evaluation; design and evaluation of primers; selection and evaluation of internal control genes; fatty acid analysis) accompanied by 6 tables which include performance of all steers fed in the study (**Table S1**), qPCR primer information (**Table S2**), validation (**Table S3 **and **S4**), qPCR performance (**Table S5**), and muscle fatty acid analysis (**Table S6**). The file also contains an additional 13 figures depicting cellular location and relationships among genes studied (**Figure S1**), relative mRNA abundance among genes (**Figure S2**), nutrient and energy intake of steers used for transcript profiling (**Figure S3**), blood concentrations of selected metabolites (**Figure S4**), diagram of approach used for selection of ICG (**Figure S5**), expression patterns of potential ICG (**Figure S6**), IPA interactions among selected ICG (**Figure S7**), longitudinal pattern of potential ICG in muscle (**Figure S8**), geNorm analysis of potential ICG (**Figure S9**), expression patterns of selected genes (**Figure S10, S11**), hierarchical clustering of gene expression patterns (**Figure S12**), and k-means clustering of gene expression patterns (**Figure S13**). For each figure a detailed legend is provided. Click here for file

Additional File 2 **The file contains Pearson correlations among all genes tested as well as blood metabolites (non-esterified fatty acids [NEFA], BUN, glucose, insulin) separated by steer type and diet combination (i.e., 4 separate sheets).** Correlations were analyzed using PROC CORR of SAS (SAS Inst. Inc. Cary, NC, release 8.0). Click here for file
