# Supplementary material for: Adipogenic and energy metabolism gene networks in longissimus lumborum during rapid post-weaning growth in Angus and Angus × Simmental cattle fed high-starch or low-starch diets
Source: BMC Genomics. 2009 Mar 31;10:142. doi: 10.1186/1471-2164-10-142 (PMC2676302; doi:10.1186/1471-2164-10-142)
Supplement: Additional File 1 — The file contains additional materials and methods (biopsy procedure; RNA extraction, PCR, and primer design and evaluation; design and evaluation of primers; selection and evaluation of internal control genes; fatty acid analysis) accompanied by 6 tables which include performance of all steers fed in the study (Table S1), qPCR primer information (Table S2), validation (Table S3 and S4), qPCR performance (Table S5), and muscle fatty acid analysis (Table S6). The file also contains an additional 13 figures depicting cellular location and relationships among genes studied (Figure S1), relative mRNA abundance among genes (Figure S2), nutrient and energy intake of steers used for transcript profiling (Figure S3), blood concentrations of selected metabolites (Figure S4), diagram of approach used for selection of ICG (Figure S5), expression patterns of potential ICG (Figure S6), IPA interactions among selected ICG (Figure S7), longitudinal pattern of potential ICG in muscle (Figure S8), geNorm analysis of potential ICG (Figure S9), expression patterns of selected genes (Figure S10, S11), hierarchical clustering of gene expression patterns (Figure S12), and k-means clustering of gene expression patterns (Figure S13). For each figure a detailed legend is provided. [file 1471-2164-10-142-S1.doc]

**Additional file 1**

***Biopsy procedure.*** Tissue was obtained from LL via needle biopsy (12 gauge core biopsy needle; Bard Magnum, C. R. Bard, Covington, GA, USA) while animals were immobilized in a cattle chute. The surgical area was clipped with fine clippers and washed with an iodine disinfectant mixture. Lidocaine-HCl (3 mL; Agri Laboratories, St. Joseph, MO, USA) was given i.m. to anesthetize the biopsy area prior to performing a 1 cm incision with a sterile scalpel blade. The first biopsy was collected from a section between the 12^th^ and 13^th^ rib on the left side of the animal. Subsequent biopsies were collected from the left side ca. 6 cm from the previous one moving towards the head. Over 0.5 g of tissue was obtained from each steer at each time point and was stored in liquid-N_2_ until RNA extraction. The incision was then closed with surgical staples (Multi-Shot Disposable Skin Stapler, 3M Medical Products; Henry Schein®, Melville, NY, USA) and iodine ointment (Povidone ointment, 10%; Henry Schein®, Melville, NY, USA) was applied to the wound. Animals were monitored daily for behavioral signs of discomfort and wound swelling or discharge. Staples typically fell-off as the wound healed and few remained by 7 d post-biopsy, when those remaining were removed. Blood was collected from the jugular vein prior to biopsies (ca. 0800 h) to isolate serum for metabolite analysis. Animals had free access to feed and consumed ca. 6 meals per day, thus minimizing the potential for postprandial effects on blood metabolite concentrations.

***RNA extraction, PCR, and primer design and evaluation.*** Biopsy tissue was weighted (~0.3-0.5 g) and immediately subjected to RNA extraction using ice-cold Trizol (Invitrogen Corp.) as described previously [1]. Genomic DNA was removed from RNA with DNase using RNeasy Mini Kit columns (Qiagen, Germany). RNA concentration was measured using a NanoDrop ND-1000 spectrophotometer (NanoDrop Technologies). The purity of RNA (A_260_/A_280_) for all samples was above 1.9. RNA quality was assessed using a 2100 Bioanalyzer (Agilent Technologies). Samples had a median RNA integrity value of 7.3 ± 0.2. A portion of the RNA was diluted to 100 mg/L using DNase/RNase free water prior to reverse transcriptase.

cDNA was synthesized using 100 ng RNA, 1 μg dT18 (Operon Biotechnologies, AL), 1 μL 10 mmol/L dNTP mix (Invitrogen Corp., CA), 1 μL random primers (Invitrogen Corp., CA), and 10 μL DNase/RNase free water. The mixture was incubated at 65 °C for 5 min and kept on ice for 3 min. A total of 6 μL of master mix composed of 4.5 μL 5X First-Strand Buffer, 1 μL 0.1 M DTT, 0.25 μL (50 U) of SuperScript^TM^ III RT (Invitrogen Corp., CA), and 0.25 μL of RNase Inhibitor (10 U, Promega, WI) was added. The reaction was performed in an Eppendorf Mastercycler® Gradient using the following temperature program: 25 °C for 5 min, 50 °C for 60 min and 70 °C for 15 min. cDNA was then diluted 1:4 (v:v) with DNase/RNase free water.

Quantitative PCR (qPCR) was performed using 4 μL diluted cDNA combined with 6 μL of a mixture composed of 5 μL 1 × SYBR Green master mix (Applied Biosystems, CA), 0.4 μL each of 10 μM forward and reverse primers, and 0.2 μL DNase/RNase free water in a MicroAmp™ Optical 384-Well Reaction Plate (Applied Biosystems, CA). Each sample was run in triplicate and a 6 point relative standard curve plus the non-template control (NTC) were used (User Bulletin #2, Applied Biosystems, CA). The reactions were performed in an ABI Prism 7900 HT SDS instrument (Applied Biosystems, CA) using the following conditions: 2 min at 50 °C, 10 min at 95 °C, 40 cycles of 15 s at 95 °C (denaturation) and 1 min at 60 °C (annealing + extension). The presence of a single PCR product was verified by the dissociation protocol using incremental temperatures to 95 °C for 15 s plus 65 °C for 15 s. Data were calculated with the 7900 HT Sequence Detection Systems Software (version 2.2.1, Applied Biosystems, CA). The final data were normalized using the geometric mean of the four most stable genes among the ones tested as internal controls, as reported previously [2].

***Design and evaluation of primers.*** Primer features for genes not reported previously by Bionaz and Loor [3, 4] are shown in Suppl. Table 1 and 2, and gene description in Suppl. Table 3. Primers were designed using Primer Express 2.0 or 3.0 with minimum amplicon size of 80 bp (when possible amplicons of 100-150 bp were chosen) and limited 3’ G+C (Applied Biosystems, CA). When possible, primers were designed to fall across exon–exon junctions. Primers were aligned against publicly available databases using BLASTN at NCBI and UCSC’s Cow (*Bos taurus*) Genome Browser Gateway [5]. Prior to qPCR primers were tested in a 20 μL PCR reaction using the same protocol described for qPCR except for the final dissociation protocol. For primer testing we used a universal reference cDNA (RNA mixture from 5 different bovine tissues) to ensure identification of desired genes. Five μL of the PCR product were run in a 2% agarose gel stained with ethidium bromide (2 μL). The remaining 15 μL were cleaned using QIAquick® PCR Purification Kit (QIAGEN) and sequenced at the Core DNA Sequencing Facility of the Roy J. Carver Biotechnology Center at the University of Illinois, Urbana-Champaign (Suppl. Table 3 and 4). Only those primers that did not present primer-dimer, a single band at the expected size in the gel, and had the right amplification product (verified by sequencing) were used for qPCR. The accuracy of a primer pairs also was evaluated by the presence of a unique peak during the dissociation step at the end of qPCR.

***Selection and evaluation of internal control genes (ICG).*** GeneSpring GX software (Agilent Technologies Inc., CA) was used initially to evaluate gene expression ratios of >10,000 genes at 0, 56, and 112 d in Angus steers fed high-starch or low-starch diets [6]. Stability (M = gene-stability measure) using geNorm [7] refers to the constancy of the expression ratio between two non-co-regulated genes among all samples tested. The more stable the expression ratio among two genes, the more likely that the genes are appropriate internal controls, i.e. two ideal control genes should have an identical expression ratio in all samples regardless of experimental conditions, cell, and/or tissue type. The lower the M value, the higher the stability. geNorm also performs an analysis to determine the utility of including more than 2 genes for normalization by calculating the pairwise variation (V) between the normalization factor (NF) obtained using n genes (best references) (NF_n_) and the NF obtained using n+1 genes (addition of an extra less stable reference gene) (NF_n+1_). A large decrease in the pairwise variation indicates that addition of the subsequent more stable gene (i.e. with lowest M value) has a significant effect and should be included for calculation of the NF [7]. Once the most stable internal reference genes are selected, the NF is calculated using the geometrical average between them to normalize qPCR data.

***Skeletal muscle tissue fatty acid analysis.*** Muscle tissue samples from Angus × Simmental steers were exhausted during RNA extraction. Angus muscle tissue lipids were extracted and methylated as reported by Loor et al. [8] and Loor and Herbein [9]. Conditions during GLC analysis and identification of fatty acids were as described in Loor and Herbein [9].

**ADDITIONAL TABLES**

**Table S1.**  Body weight, average daily gain (ADG), dry matter intake, NE_G_ intake, residual feed intake (RFI), feed efficiency, marbling score, back fat thickness, and *longissimus lumborum* muscle depth in all steers fed in this study: Angus and Angus × Simmental (A×S) steers a high-starch (HiS) or low-starch (LoS) diet during a 112 d growing phase.

|  | Treatments | | | | |  |  |  | | |
| --- | --- | --- | --- | --- | --- | --- | --- | --- | --- | --- |
|  | HiS | |  | LoS | |  |  | *P* value | | |
| Item | Angus | A×S |  | Angus | A×S | SEM |  | Diet | Steer type | Diet × Steer type |
| n = | 9 | 6 |  | 8 | 6 |  |  |  |  |  |
| Body weight (kg) |  |  |  |  |  |  |  |  |  |  |
| Initial | 169 | 179 |  | 155 | 183 | 13 |  | 0.69 | 0.11 | 0.46 |
| d 56 | 247 | 251 |  | 218 | 257 | 12 |  | 0.41 | 0.12 | 0.20 |
| Final | 342 | 351 |  | 315 | 363 | 20 |  | 0.68 | 0.12 | 0.29 |
| ADG (kg/d) |  |  |  |  |  |  |  |  |  |  |
| 0 to 56 d | 1.43 | 1.35 |  | 1.15 | 1.35 | 0.05 |  | 0.04 | 0.35 | 0.04 |
| 0 to 112 d | 1.54 | 1.52 |  | 1.42 | 1.60 | 0.08 |  | 0.79 | 0.28 | 0.20 |
| Dry matter intake (kg/d) |  |  |  |  |  |  |  |  |  |  |
| 0 to 112 d | 6.97 | 6.68 |  | 6.96 | 8.45 | 0.35 |  | 0.01 | 0.06 | 0.01 |
| NE_G_ intake (Mcal/d)^1^ |  |  |  |  |  |  |  |  |  |  |
| 0 to 112 d | 9.82 | 9.40 |  | 8.15 | 9.90 | 0.47 |  | 0.16 | 0.11 | 0.01 |
| Residual feed intake^2^ | -0.79 | -1.87 |  | 0.48 | 1.43 | 0.62 |  | 0.0001 | 0.91 | 0.07 |
| Feed efficiency (kg/kg)^3^ |  |  |  |  |  |  |  |  |  |  |
| 0 to 112 d | 0.22 | 0.23 |  | 0.20 | 0.19 | 0.01 |  | 0.005 | 0.81 | 0.22 |
| Day 112 ultrasound |  |  |  |  |  |  |  |  |  |  |
| Marbling score | 4.44 | 4.01 |  | 4.58 | 4.23 | 0.19 |  | 0.31 | 0.03 | 0.85 |
| Back fat (mm) | 0.19 | 0.16 |  | 0.16 | 0.14 | 0.02 |  | 0.33 | 0.33 | 0.62 |
| Muscle depth (mm) | 55.4 | 52.0 |  | 51.4 | 52.2 | 2.2 |  | 0.36 | 0.51 | 0.31 |

^1^ Estimated from actual dry matter intake (kg/d) × calculated NE_G_ (1.19 or 1.43 Mcal/kg diet dry matter for LoS or HiS).

^2^ Residual feed intake calculated by regression [10] of actual dry matter intake against average metabolic body weight (body weight ^0.75^) and ADG.

^3^ADG/feed intake.

**Table S2.** GenBank accession number, hybridization position, sequence and amplicon size of primers for *Bos* *taurus* used to analyze gene expression by qPCR.

| **Accession #** | **Gene** | **Primers^1^** | **Primers (5’-3’)^2^** | **bp^3^** |
| --- | --- | --- | --- | --- |
| BT020877 | *EDG1* | F.581  R.690 | TGCGGGAAGGGAGTATGTTT  GCTCCCATTGTGGAGTTTCATC | 110 |
| BC102935 | *RBMS2* | F.514  R.623 | GCAAGAACTGGAGGGAATGC  TGGACTCCATCCTGGCAAAG | 110 |
| BC112619 | *C20ORF196* | F.9  R.98 | AGCTACTGCCCGGTGGACTAT  TAAGCTGATGGCAGGTCCAAA | 90 |
| BC120279 | *ARRDC1* | F.394  R.495 | CGACACACCACGTTTTTCCA  ACATTGGGTTGCTCGATGTCT | 102 |
| BT030480 | *ACTB* | F.258  R.406 | ACCAACTGGGACGACATGGA  GTCTCGAACATGATCTGGGTCAT | 149 |
| BC108231 | *RPS15A* | F.31  R.131 | GAATGGTGCGCATGAATGTC  GACTTTGGAGCACGGCCTAA | 101 |
| AK074976 | *MTG1* | F.696  R.796 | CTTGGAATCCGAGGAGCCA  CCTGGGATCACCAGAGCTGT | 101 |
| BQ676558 | *UXT* | F.323  R.423 | TGTGGCCCTTGGATATGGTT GGTTGTCGCTGAGCTCTGTG | 101 |
| BC108138 | *ACLY* | F.2287  R.2390 | GTTCTCCTCCGAGGTCCAGTT CAAACACTCCAGCCTCCTTCA | 104 |
| DN525902 | *G6PD* | F.426  R.532 | ACCAGGGCACACAGACCAA TTCCAGCCTGTCTGGCTCAT | 107 |
| AY574999 | *INSR* | F.245  R.328 | CCCTTCGAGAAAGTGGTGAACA AGCCTGAAGCTCGATGCGATAG | 84 |
| CR551751 | *IRS1* | F.73  R.184 | TGTTGACTGAACTGCACGTTCT CATGTGGCCAGCTAAGTCCTT | 112 |
| BC109597 | *MDH2* | F.597  R.696 | GTCGCAGAGCTGAAGGATTTG GGGTGCACTGGGAGATCAAG | 100 |
| EE372759 | *PRKAA1* | F.524  R.624 | GGCATTTGGGAATTAGAAGTCAA  CGGGTTTACAACCTTCCATTCA | 101 |
| CK777791 | *PRKAA2* | F.236  R.352 | GGATGGCTAGCAACCAAGATG  CCTCCCCTGATCACCTTTGTCT | 117 |
| BC120057 | *SLC2A4* | F.1582  R.1682 | AGGCCTACCTCAGCGGTGA  CACGTTCTCGCCTTTCCAG | 101 |

^1^ Primer direction (F – forward; R – reverse) and hybridization position on the sequence.

^2^ Exon-exon junctions are underlined.

^3^ Amplicon size in base pair (bp).

**Table S3.** Sequencing results of PCR products from primers of genes designed for this experiment. Best hits using BLASTN (http://www.ncbi.nlm.nih.gov) are shown. Similar information for remaining genes was reported previously [2].

| **Gene** | **Sequence** |
| --- | --- |
| *ACLY* | CTGGACTGTGCCACCAGGCTTCTGAAACCTGCAGTTGCCAAGAACCAGGCCTTGAAGGAGGCTGGAGTGCTTTGGA |
| *ARRDC1* | GCCCCCTGAACCTGAACAGCATCCCAGACATCGAGCAACCACAATGTAG |
| *C20ORF196* | GGGTAGCCAGACAGGAGGAGAGCAATGCTTTGGACCTGCCATCAGCTTAA |
| *EDG1* | TGTCAGCCTCCTGGCATCGCCATTGAGCGCTACATCACCATGCTGAAGATGAAACTCCACAATGAGGAGCA |
| *G6PD* | CTCCTGGCTGTCCCGACTGTCTATGAGGCTGTCACCAAAAACATCCATGAGACCTGCATGAGCCAGACAGGCTGGAAAA |
| *INSR* | AGCTGCGGTCTATCTCCGGCCTGCGTCACTTTACTGGCTATCGCATCGAGCTATCAGGCTACG |
| *IRS1* | ATCAGGCAGAAAAGCACTGTGACACCAGAACAATGAGTCTGCATAAACTTCATCTTCAACCTTAAGGACTTAGCTGGCCAACATGGAA |
| *MDH2* | GCAACGACTACGTCCGGTCATCGGGCGGGCCACGCTGGGGAAAACCATCATCCCCTTGGATCTCCCAGTGCACCCAGA |
| *PRKAA1* | AACCGCCAGTCATACTATGGCAGAGTTTGTAGAGCAATTAAACAGCTGGATTATGAATGGAAGGTTGTAACACCACGAC |
| *PRKAA2* | CTAGAGACCGAGAGATTCAAACTAAGCCCATCAGCCACTAAGGATCAAACAATAAACAAAGACCAATAGGTGATCAGGGGAGGAT |
| *RBMS2* | CCAGGTTATCTCCACTAGAATCCTTCGAGACACCAGATGGGACCAGCAGAGGGGTTGGCTTTGCCAGGATGGAGTCCAA |

**Table S4.** Sequencing results of genes using BLASTN from NCBI [11] against nucleotide collection (nr / nt) with total score.

| **Gene Name** | **Best hit in NCBI** | **Score** |
| --- | --- | --- |
| *EDG1* | Bos taurus endothelial differentiation, sphingolipid G-protein-coupled receptor, 1 | 111 |
| *RBMS2* | Bos taurus RNA binding motif, single stranded interacting protein 2 (RBMS2) | 132 |
| *C20ORF196* | Bos taurus hypothetical protein LOC787583 (LOC787583) | 79.8 |
| *ARRDC1* | Bos taurus hypothetical protein LOC786098 (MGC142598) | 79.8 |
| *G6PD* | Bos taurus glucose-6-phosphate dehydrogenase (G6PD), mRNA | 120 |
| *INSR* | Bos taurus insulin receptor mRNA, partial cds | 82.4 |
| *IRS1* | Homo sapiens insulin receptor substrate 1 (IRS1), mRNA | 120 |
| *MDH2* | Bos taurus mitochondrial malate dehydrogenase 2, NAD (MDH2) mRNA, partial cds; nuclear gene for mitochondrial product | 77 |
| *PRKAA1* | Bos taurus similar to Protein kinase, AMP-activated, alpha 1 catalytic subunit (LOC782795), partial mRNA | 91.5 |
| *PRKAA2* | Homo sapiens protein kinase, AMP-activated, alpha 2 catalytic subunit (PRKAA2), mRNA | 60.8 |

**Table S5.** qPCR performance among the 31 genes measured in skeletal muscle.

| **Gene** | **Median Ct^1^** | **Median ∆Ct^2^** | **Slope^3^** | **(R^2^)^4^** | **Efficiency^5^** |
| --- | --- | --- | --- | --- | --- |
| *ACACA* | 24.1 | 2.9 | -3.23 | 0.995 | 2.04 |
| *ACLY* | 24.8 | 3.8 | -2.91 | 0.994 | 2.21 |
| *ACSL1* | 20.4 | -0.1 | -3.07 | 0.995 | 2.12 |
| *AGPAT1* | 23.5 | 3.0 | -3.18 | 0.997 | 2.06 |
| *CD36* | 20.5 | -0.4 | -3.04 | 0.995 | 2.13 |
| *DGAT1* | 25.1 | 3.7 | -2.91 | 0.997 | 2.21 |
| *DGAT2* | 21.9 | 0.7 | -2.89 | 0.996 | 2.22 |
| *FABP4* | 22.4 | 1.5 | -2.97 | 0.993 | 2.17 |
| *FADS2* | 22.6 | 1.7 | -3.12 | 0.996 | 2.09 |
| *FASN* | 20.5 | 0.2 | -3.29 | 0.992 | 2.01 |
| *G6PD* | 22.6 | 1.8 | -3.23 | 0.990 | 2.04 |
| *GLUT4* | 19.9 | -1.2 | -3.09 | 0.996 | 2.11 |
| *GPAM* | 23.9 | 3.1 | -3.23 | 0.998 | 2.04 |
| *INSIG1* | 22.2 | 2.0 | -3.01 | 0.990 | 2.15 |
| *INSR* | 23.1 | 0.9 | -2.93 | 0.992 | 2.20 |
| *IRS1* | 22.1 | -1.0 | -3.03 | 0.995 | 2.14 |
| *LPIN1* | 20.1 | 0.3 | -3.09 | 0.994 | 2.11 |
| *LPIN2* | 26.2 | 5.8 | -3.28 | 0.993 | 2.02 |
| *LPIN3* | 24.5 | 3.7 | -3.11 | 0.997 | 2.10 |
| *MDH2* | 18.3 | -2.9 | -3.06 | 0.996 | 2.12 |
| *PPARD* | 22.7 | 2.0 | -3.14 | 0.993 | 2.08 |
| *PPARG* | 25.1 | 3.6 | -2.76 | 0.997 | 2.30 |
| *PPARGC1A* | 21.6 | 0.4 | -2.96 | 0.996 | 2.18 |
| *PPARGC1B* | 22.7 | 1.2 | -3.00 | 0.997 | 2.15 |
| *PRKAA1* | 23.6 | 2.6 | -2.97 | 0.994 | 2.17 |
| *PRKAA2* | 20.5 | -0.2 | -3.02 | 0.995 | 2.14 |
| *SCAP* | 22.4 | 1.3 | -2.81 | 0.995 | 2.27 |
| *SCD* | 19.2 | -1.3 | -3.17 | 0.997 | 2.07 |
| *SLC27A1* | 22.7 | 1.5 | -3.01 | 0.993 | 2.15 |
| *SREBF1* | 22.8 | 1.3 | -3.38 | 0.997 | 1.98 |
| *THRSP* | 23.4 | 3.1 | -3.11 | 0.991 | 2.10 |

^1^ The median is calculated considering all time points and all steers.

^2^ The median of ∆Ct is calculated as [Ct gene – geometrical mean of Ct internal controls] for each time point and each steer.

^3^ Slope of the standard curve.

^4^ R^2^ stands for the coefficient of determination of the standard curve.

^5^ Efficiency is calculated as [10^(-1 / Slope)^].

**Table S6.**  Fatty acid composition (g/100 g total fatty acids) of *longissimus lumborum* from Angus steers fed a high-starch (HiS, n = 3/type) or low-starch (LoS, n = 3/type) diet during a 112 d growing phase.

|  | Treatments | | | | | | |  |  |  |  |  |  |
| --- | --- | --- | --- | --- | --- | --- | --- | --- | --- | --- | --- | --- | --- |
|  | HiS | | |  | LoS | | |  |  |  | P-value | | |
| **Fatty acid** | **0** | **56** | **112** |  | **0** | **56** | **112** |  | **SEM** |  | **Diet** | **Time** | **D × T** |
| 12:0 | 0.24 | 0.17 | 0.14 |  | 0.29 | 0.16 | 0.08 |  | 0.026 |  | 0.77 | <.0001 | 0.10 |
| 14:0 | 2.51 | 2.19 | 2.32 |  | 2.58 | 2.36 | 2.43 |  | 0.188 |  | 0.47 | 0.34 | 0.96 |
| cis9-14:1 | 0.40 | 0.47 | 0.53 |  | 0.37 | 0.48 | 0.51 |  | 0.055 |  | 0.80 | 0.05 | 0.95 |
| 15:0 | 0.61 | 0.54 | 0.56 |  | 0.58 | 0.39 | 0.34 |  | 0.052 |  | 0.02 | 0.01 | 0.19 |
| 16:0 | 22.30 | 25.18 | 25.86 |  | 22.17 | 27.79 | 29.60 |  | 0.669 |  | 0.01 | <.0001 | 0.02 |
| trans9-16:1 | 0.24 | 0.19 | 0.18 |  | 0.24 | 0.20 | 0.16 |  | 0.032 |  | 0.94 | 0.10 | 0.81 |
| trans11-16:1 | 0.51 | 0.31 | 0.31 |  | 0.59 | 0.59 | 0.56 |  | 0.064 |  | 0.01 | 0.11 | 0.15 |
| cis9-16:1 | 1.55 | 2.55 | 2.61 |  | 1.54 | 2.52 | 2.96 |  | 0.189 |  | 0.56 | <.0001 | 0.49 |
| cis11-16:1 | 0.16 | 0.21 | 0.22 |  | 0.22 | 0.14 | 0.16 |  | 0.029 |  | 0.31 | 0.75 | 0.06 |
| 17:0 | 1.03 | 1.43 | 1.60 |  | 1.05 | 0.77 | 0.89 |  | 0.108 |  | 0.00 | 0.17 | 0.00 |
| cis9-17:1 | 0.40 | 0.25 | 0.17 |  | 0.43 | 0.15 | 0.06 |  | 0.032 |  | 0.04 | <.0001 | 0.07 |
| 18:0 | 14.37 | 13.85 | 14.52 |  | 15.15 | 15.08 | 14.79 |  | 0.455 |  | 0.10 | 0.75 | 0.49 |
| trans6-9-18:1 | 0.83 | 0.56 | 0.49 |  | 0.79 | 0.49 | 0.51 |  | 0.183 |  | 0.77 | 0.17 | 0.41 |
| trans10-18:1 | 0.91 | 1.51 | 1.21 |  | 1.24 | 1.35 | 1.17 |  | 0.088 |  | 0.73 | 0.01 | 0.83 |
| trans11-18:1 | 0.66 | 0.36 | 0.45 |  | 0.68 | 0.42 | 0.46 |  | 0.061 |  | 0.61 | <.0001 | 0.83 |
| trans12-18:1 | 0.05 | 0.05 | 0.08 |  | 0.08 | 0.06 | 0.07 |  | 0.015 |  | 0.56 | 0.28 | 0.65 |
| trans13-18:1 | 0.47 | 0.24 | 0.19 |  | 0.39 | 0.41 | 0.11 |  | 0.073 |  | 0.96 | 0.01 | 0.16 |
| cis9-18:1 | 23.86 | 30.33 | 33.75 |  | 23.21 | 29.39 | 34.40 |  | 1.238 |  | 0.76 | <.0001 | 0.79 |
| cis11-18:1 | 1.33 | 1.31 | 1.17 |  | 1.40 | 0.96 | 0.82 |  | 0.046 |  | 0.00 | <.0001 | <.0001 |
| cis12-18:1 | 0.18 | 0.15 | 0.12 |  | 0.24 | 0.18 | 0.17 |  | 0.020 |  | 0.04 | 0.01 | 0.61 |
| cis13-18:1 | 0.18 | 0.34 | 0.34 |  | 0.22 | 0.20 | 0.22 |  | 0.024 |  | 0.01 | 0.01 | 0.00 |
| cis15-18:1 | 0.11 | 0.07 | 0.07 |  | 0.09 | 0.08 | 0.08 |  | 0.008 |  | 0.64 | 0.01 | 0.15 |
| **18:2 isomers** |  |  |  |  |  |  |  |  |  |  |  |  |  |
| trans9,trans12 | 0.05 | 0.09 | 0.05 |  | 0.09 | 0.05 | 0.01 |  | 0.018 |  | 0.35 | 0.07 | 0.06 |
| cis9,trans12 | 0.72 | 0.91 | 0.59 |  | 0.96 | 0.66 | 0.14 |  | 0.188 |  | 0.40 | 0.03 | 0.17 |
| trans9,cis12 | 0.63 | 0.77 | 0.48 |  | 0.86 | 0.48 | 0.17 |  | 0.147 |  | 0.38 | 0.02 | 0.12 |
| cis9,cis12 | 13.53 | 9.55 | 7.45 |  | 13.55 | 8.49 | 5.67 |  | 0.852 |  | 0.22 | <.0001 | 0.57 |
| cis9,trans11 | 0.33 | 0.21 | 0.19 |  | 0.25 | 0.24 | 0.25 |  | 0.046 |  | 0.91 | 0.23 | 0.31 |
| cis11,trans13 | 0.05 | 0.04 | 0.03 |  | 0.06 | 0.03 | 0.02 |  | 0.005 |  | 0.48 | <.0001 | 0.09 |
| cis11,cis13 | 0.08 | 0.04 | 0.03 |  | 0.08 | 0.07 | 0.03 |  | 0.011 |  | 0.53 | 0.01 | 0.30 |
| 18:3n-6 | 0.11 | 0.10 | 0.08 |  | 0.11 | 0.08 | 0.06 |  | 0.009 |  | 0.05 | 0.01 | 0.34 |
| 18:3n-3 | 0.52 | 0.17 | 0.11 |  | 0.47 | 0.27 | 0.22 |  | 0.033 |  | 0.06 | <.0001 | 0.04 |
| 20:0 | 0.48 | 0.28 | 0.16 |  | 0.39 | 0.28 | 0.19 |  | 0.069 |  | 0.73 | 0.01 | 0.64 |
| 20:2n-6 | 0.24 | 0.19 | 0.14 |  | 0.26 | 0.17 | 0.09 |  | 0.021 |  | 0.34 | <.0001 | 0.23 |
| 20:3n-6 | 1.10 | 0.69 | 0.53 |  | 1.11 | 0.67 | 0.39 |  | 0.083 |  | 0.46 | <.0001 | 0.63 |
| 20:4n-6 | 4.78 | 2.49 | 1.84 |  | 4.87 | 2.49 | 1.30 |  | 0.326 |  | 0.58 | <.0001 | 0.59 |
| 20:5n-3 | 1.02 | 0.27 | 0.10 |  | 0.90 | 0.36 | 0.19 |  | 0.104 |  | 0.79 | <.0001 | 0.50 |
| 22:0 | 1.09 | 0.51 | 0.22 |  | 0.81 | 0.59 | 0.37 |  | 0.186 |  | 0.91 | 0.01 | 0.47 |
| 22:1 | 0.20 | 0.15 | 0.11 |  | 0.21 | 0.16 | 0.07 |  | 0.029 |  | 0.81 | 0.01 | 0.62 |
| 22:4n-6 | 0.24 | 0.24 | 0.24 |  | 0.25 | 0.22 | 0.16 |  | 0.025 |  | 0.24 | 0.19 | 0.13 |
| 22:5n-6 | 0.04 | 0.04 | 0.05 |  | 0.04 | 0.04 | 0.03 |  | 0.005 |  | 0.07 | 0.74 | 0.08 |
| 22:5n-3 | 1.12 | 0.53 | 0.30 |  | 1.15 | 0.56 | 0.27 |  | 0.066 |  | 0.84 | <.0001 | 0.86 |
| 22:6n-3 | 0.17 | 0.07 | 0.03 |  | 0.16 | 0.07 | 0.04 |  | 0.017 |  | 0.95 | <.0001 | 0.92 |
| 24:0 | 0.28 | 0.12 | 0.06 |  | 0.20 | 0.15 | 0.09 |  | 0.046 |  | 0.89 | 0.01 | 0.36 |
| 12:0-16:0 | 25.49 | 28.05 | 28.88 |  | 25.37 | 30.74 | 32.57 |  | 0.802 |  | 0.02 | <.0001 | 0.06 |
| **Desaturase indexes** | |  |  |  |  |  |  |  |  |  |  |  |  |
| cis9-14:1/14:0 | 0.15 | 0.21 | 0.22 |  | 0.15 | 0.20 | 0.21 |  | 0.016 |  | 0.64 | <.0001 | 0.98 |
| cis9-16:1/16:0 | 0.07 | 0.10 | 0.10 |  | 0.07 | 0.09 | 0.10 |  | 0.001 |  | 0.58 | <.0001 | 0.56 |
| cis9-18:1/18:0 | 1.68 | 2.20 | 2.31 |  | 1.56 | 1.99 | 2.37 |  | 0.133 |  | 0.47 | <.0001 | 0.58 |
| 20:4/cis9,cis12-18:2 | 0.35 | 0.25 | 0.24 |  | 0.36 | 0.29 | 0.22 |  | 0.016 |  | 0.61 | <.0001 | 0.28 |
| CLA/trans11-18:1 | 0.63 | 0.79 | 0.48 |  | 0.42 | 0.62 | 0.64 |  | 0.168 |  | 0.62 | 0.53 | 0.51 |
| total trans-18:1 | 2.95 | 2.72 | 2.43 |  | 3.16 | 2.73 | 2.33 |  | 0.215 |  | 0.83 | 0.01 | 0.56 |
| total cis-18:1 | 26.10 | 32.64 | 35.89 |  | 24.72 | 30.37 | 35.25 |  | 1.803 |  | 0.49 | <.0001 | 0.80 |
|  |  |  |  |  |  |  |  |  |  |  |  |  |  |
|  |  |  |  |  |  |  |  |  |  |  |  |  |  |
| **Table S6 continued** | | | |  |  |  |  |  |  |  |  |  |  |
|  |  |  |  |  |  |  |  |  |  |  |  |  |  |
|  |  |  |  |  |  |  |  |  |  |  |  |  |  |
| total 18:2 | 15.03 | 11.41 | 8.68 |  | 15.37 | 9.58 | 5.89 |  | 1.090 |  | 0.16 | <.0001 | 0.35 |
| total 18:3 | 0.62 | 0.26 | 0.19 |  | 0.58 | 0.36 | 0.28 |  | 0.039 |  | 0.15 | <.0001 | 0.17 |
| total CLA | 0.48 | 0.30 | 0.26 |  | 0.37 | 0.32 | 0.28 |  | 0.054 |  | 0.66 | 0.02 | 0.41 |
| total 20-carbon | 10.24 | 5.25 | 3.58 |  | 9.99 | 5.54 | 3.03 |  | 1.103 |  | 0.90 | <.0001 | 0.80 |
| total fatty acids (mg/g) | 4.12 | 6.36 | 9.02 |  | 4.26 | 8.05 | 10.25 |  | 0.937 |  | 0.15 | <.0001 | 0.72 |

**Figure S1.** Cellular location and currently known relationships among selected genes. Networks were generated using Ingenuity Pathway Analysis®. Arrows denote direct (solid lines) or indirect (dotted lines) interactions among genes. Interactions include: E, expression; PD, protein-DNA binding; PR, protein-RNA binding; PP, protein-protein binding; RB, regulation of binding; T, transcription; A, activation; LO, localization; TR, translocation; P, phosphorylation/dephosphorylation.

**Figure S2.** Relative % mRNA abundance among genes in LL tissue of Angus and Angus × Simmental (A×S) steers (n = 6/type) fed a high-starch (HiS; n = 3/type) or low-starch (LoS; n = 3/type) diet during a 112 d growing phase. The relative % mRNA was calculated as described previously [3].

**Figure S3.** Daily dry matter (top panel) and energy (bottom panel) intake during the growing phase in Angus or Angus × Simmental steers (n = 6/type) fed a high-starch (HiS, n = 3/type) or low-starch (LoS, n = 3/type) diet. Statistical effects for dry matter intake were: Diet, P = 0.07; Steer type, P = 0.04; Time, P < 0.01; Steer type × diet, P = 0.05; diet × time, P < 0.01; steer type × time, P = 0.02; Diet × steer type × time, P < 0.01. Statistical effects for energy intake were: Diet, P = 0.41; Steer type, P = 0.06; Time, P < 0.01; Steer type × diet, P = 0.07; Diet × time, P < 0.01; Steer type × time, P = 0.02; Diet × steer type × time, P < 0.01.

**Figure S4.** Blood serum concentrations of NEFA, BHBA, and BUN during the growing phase in Angus or A×S steers (n = 6/type) fed a high-starch (HiS, n = 3/type) or low-starch (LoS, n = 3/type) diet. Asterisks denote significant (P < 0.05) effects of time.

**Figure S5.** Flow diagram of selection criteria used to identify suitable ICG for qPCR normalization.

**Figure S6.** Microarray expression patterns (*n*-fold-scale) of potential ICG selected for co-regulation analysis using IPA. Samples were from the study of Graugnard et al. [6]. Yellow and orange lines depict the pattern of stably-expressed genes (i.e., selected ICG) across all samples. In left panel, red and green lines denote the expression pattern of *ACTB* and *GAPDH*. In right panel, expression patterns of cyclophilin isoforms are shown. *PPIA* has been previously used as ICG in bovine muscle [12, 13]. Image generated with GeneSpring GX (Agilent Technologies).

**Figure S7.** Interactions and cellular location of genes tested as ICG. Networks were developed using Ingenuity Pathway Analysis®. Solid lines denote direct interactions and dotted lines indirect interactions. Encircled are the tested ICG.

**Figure S8.** Relative gene expression patterns of potential ICG in *longissimus* *lumborum* muscle of Angus steer calves fed a high-starch (HiS) corn-based diet or a low-starch fiber-based diet (LoS) during the growing phase (0 to 112 d) [6]. *Time effect *P* < 0.05. **Time and time × treatment effect *P* < 0.05.

**Figure S9.** Average stability (M = gene stability measure) of expression ratio values of remaining genes tested during pairwise comparison (panel A). Stability values are reported as stepwise exclusion of the least stable control gene. *C20ORF196*, *ARRDC1*, and *EDG1* were the least stable genes, while *RBMS2*, *RPS15A*, *UXT*, and *MTG1* were the most stable. Optimal number of internal reference genes for qPCR normalization (panel B). Y-axis, pairwise variation V (V_n/n+1_) between the normalization factors NF_n_ and NF_n+1_. X-axis, comparison between the use of n or n+1 genes to calculate the normalization factor, i.e., V2/3 is the comparison between the use of 3 vs. 2 ICG to calculate the normalization factor. Analysis suggested that use of 6 ICG would provide the optimal NF. However, the stability of expression using 3 or 4 ICG is below the previously-define acceptable threshold of 0.10 [2].

**Figure S10.** mRNA expression patterns of genes associated with ligand-induced activation of fatty acid oxidation and energy generation (*PPARD, PPARGC1B*), mitochondrial biogenesis (*PPARGC1A*), and the catalytic subunits of 5’-AMP-activated protein kinase (*PRKAA1*, *PRKAA2*), and diacylglycerol formation (*LPIN1*). Fold-change expression during the growing phase is expressed relative to day 0. Pooled SEM: *PPARD*, 0.2; *PPARGC1A*, 0.3; *LPIN1*, 0.2; *PRKAA1*, 0.3; *PRKAA2*, 0.2. Asterisks denote (P < 0.05): *Time effect; **Diet effect; ***Steer type effect; ****Tendency (P = 0.10) for diet × steer type × day interaction; *****Tendency (P = 0.12) for diet × steer type interaction. Superscripts denote differences (P < 0.06) among treatments at specific time points.

**Figure S11.** mRNA expression patterns of genes associated with fatty acid translocation (*CD36*), diacylglyerol formation (*LPIN3*), fatty acid uptake (*SLC27A1*), acylation of fatty acids to diacylglycerol and formation of TAG (*DGAT1*), and regulation of SREBP activity (*SCAP*). Fold-change expression during the growing phase is expressed relative to day 0. Pooled SEM: *CD36*, 0.2; *LPIN3*, 0.2; *SLC27A1*, 0.1; *DGAT1*, 0.1; *SCAP*, 0.2. Asterisks denote (P < 0.05): *Time effect; **Diet effect; ***Steer type effect; ****Diet × steer type × day interaction; &Tendency (P = 0.13) for diet × steer type × day interaction. Superscripts denote differences (P < 0.06) among treatments at specific time points.

**Figure** **S12.** Hierarchical clustering analysis of gene expression patterns using Genesis software [14] for each steer type and diet combination on d 56 and 112 of the experiment relative to d 0. For all panels, X-axis corresponds to Angus steers fed HiS day 56 (HA56), HiS day 112 (HA112), LoS day 56 (LA56), and LoS day 112 (LA112). Similarly, Angus × Simmental fed HiS day 56 (HAS56), HiS day 112 (HAS112), LoS day 56 (LAS56), and LoS day 112 (LAS112). White dots denote peak gene expression for each specific gene.

**Figure S13.** k-means clustering analysis of expression patterns on d 56 and 112 of the experiment relative to d 0. Genes within each cluster are shown. For all panels, X-axis corresponds to Angus steers fed HiS day 56 (HA56), HiS day 112 (HA112), LoS day 56 (LA56), and LoS day 112 (LA112). Similarly, Angus × Simmental fed HiS day 56 (HAS56), HiS day 112 (HAS112), LoS day 56 (LAS56), and LoS day 112 (LAS112). Genesis software [14] was used to determine the most appropriate number of clusters using figure of merit analysis.

**References**
